# Making microbes matter: storytelling’s potential to make antibiotic resistance real and relevant to the public

**DOI:** 10.1038/s44259-023-00012-5

**Published:** 2023-09-08

**Authors:** Becky McCall, Laura Shallcross, Michael Wilson, Andrew Hayward

**Affiliations:** 1 Institute of Health Informatics, University College London, London, UK; 2 School of Design and Creative Arts, Loughborough University, Loughborough, UK; 3 Institute of Epidemiology and Health Care, University College London, London, UK

**Keywords:** Public health, Health care

## Abstract

This commentary discusses how digital storytelling may help people engage with the threat of bacterial antimicrobial resistance (AMR) including in relation to antibiotic use. The range of public health communication campaigns of recent decades have had variable impacts and there remains scope to develop novel ways to make the complex issue of AMR more real and relevant to the public. Here, we propose that structured storytelling, in particular digital storytelling, may offer a more self-reflective and meaningful approach to such communication. Subtle and overt socio-cultural determinants are at play in the public’s interpretation and response to the threat of AMR, whether addressed within or without of a medical consultation, and storytelling is framed as a way of negotiating the nuances of authentic personal experience, with the person–patient storyteller at its heart.

## Introduction

The human relationship with microbes often adopts a war-like, science fiction mantle of urgency peppered with the terms, ‘superbug’ and ‘apocalypse’ and as such has filled many pages and screens across both fictional and non-fictional media over recent decades. But characterising antimicrobial resistance (AMR) as the Stars Wars of microbiology might be better replaced with true-to-life personal storytelling that engages and nurtures an understanding among the public that sits closer to home.

And, just as fear-invoking language can often fails to resonate and nurture real, balanced and personal meaning, so big statistics, in our information-flooded age, may also be wide of the mark. A case in point is the 4.95 million global deaths documented to be associated with bacterial AMR in 2019, which importantly quantifies and projects the scale of the AMR crisis^1^. But statistics alone, by the nature of reducing effects to numbers, do not necessarily impart meaning that resonates on a personal level, nor do they express emotion or help an individual identify and empathise with a character or provide a means of richly contextualised engagement with AMR experiences that may change intention and possibly behaviour^2^. Both impactful statistics and meaningful narratives are needed to jostle with the torrent of other humanitarian crises—health and other, for the attention of the people’s hearts and minds.

AMR is a complex, and vast global health issue that requires an intersectoral and interdisciplinary approach to both research and solutions—it is extensive in scope, spanning veterinary, farming, environmental and human health domains. Our focus in this article is directed towards storytelling as befits AMR within the human health arena mostly because it chimes with our combined experience and places some boundaries on an extensive field of research.

Of note, this article discusses one possible interpretation of why some AMR public communication efforts have limited effect and lays out one potential option—storytelling—a structured but subtler and more self-reflective approach to communication that might reap potential benefit when used alongside other communication methods, in the effort to make AMR more real and relevant to the public.

## Certainty of now—cognitive and social bias

The problem is underpinned by temporal and spatial considerations. There is value in the sureness of *now*, not the uncertainty of a distant future. It has been suggested that evolution determines that humans have a cognitive bias to respond to an immediate and definite threat (think fight, flight or be eaten), and ignore longer term, complex and only possible ones^3^. Hence, we might cognitively battle with the natural instinct to use an antibiotic to alleviate an annoying cough and cold now and deal with the distant threat of antibiotic resistance later—and as true as this is for adults, it is even more so for the parents or caregivers of children especially young ones—who harbour a constant stream of different viral and bacterial infections^4^.

While this cognitive bias manifests in people’s responses to using antibiotics, there is a close comparison with the public’s response to the impact of climate change. There are many parallels, for example, poorer countries are hardest hit with both AMR and climate change, and both wreak havoc across the lives of humans, animals and environment globally. We read of polar ice caps melting, floods destroying livelihoods, wildfires wrecking homes, most often thousands of miles away (hence the spatial element related to the impact of both crises). Similar to AMR, climate change also has landmark numbers including, for example, a target rise of 1.5 °C in average global temperature over pre-industrial levels; or that 200 million people globally are projected to live beneath the tideline in 70 years^5^. But, like AMR, although most people believe it is a real phenomenon and has the potential to devastate lives in the millions, they do not perceive it as a problem that will change their daily life, on their doorstep, here and now.

From a sociological standpoint, priority is given to proximal individual benefit over distal societal risk—or temporal myopia, a term adopted by AMR sociologist, Alex Broom. AMR-related temporal myopia can be interpreted in many ways, but focussing on the clinical setting, AMR is implicated in a clinician’s preference for immediate infection management with antimicrobial therapy to avoid an often, low-risk of patient worsening, in the nurturing of patient satisfaction, and in meeting social norms and hierarchical expectations around infection management. In light of apparent immediate gains, the value placed on the threat of future bio-insecurity is minimised^6^.

## AMR or climate change—nuance underpins the communication of complexity

But, directly telling people what they should and should not do, in light of a complex threat, whether AMR, climate change or other, is rarely as productive as intended, often because uptake and outcomes of prevention education are mediated by social and cultural inequities and differences that underpin and shape people’s responses^7^. To improve effectiveness, communication might insightfully incorporate such nuance into its approach.

Also, among the myriad other influences on antibiotic use, are both the clinical needs of the person-patient, as well as the perceptions related to the need for antibiotics on behalf of patient and doctor—for example, a young, healthy adult will rarely visit the GP, while a person with chronic obstructive pulmonary disease (COPD) relies on antibiotics to stay out of hospital.

Risk perception is another central component of both AMR and similarly climate change that muddies the water of communication. With climate change, risk communication requires the relaying of information across the spectrum of certainty to uncertainty associated with climate change and its impacts, as well as giving consideration to the roles of different worldviews, for example, a fatalist believes climate change is outside of human control and disengages with any communication; while a pro-environmentalist stance might perceive greater personal threat, and more willingness to engage. Greater attention to both understanding risk perceptions among different population groups, and accordingly sculpting communication to reflect this, might yield return in public mobilisation and action around AMR.

For at least two decades, public health organisations and governments across the world have run public health information campaigns, largely focussing on the need to reduce unnecessary antibiotic use, or highlighting that they do not work against viruses, for instance. Yet, evaluations of such campaigns have shown that having greater knowledge was often not associated with improved antibiotic-related behaviours^8^. A case in point being the evaluation of a European Union (EU) campaign that ran annually from 2008 to 2013, and which found that 41% of the target public still did not know that antibiotics were ineffective for colds and flu^9^.

Among the possible reasons for the poor impact of these campaigns and others, is that the knowledge and messages presented lack meaning, resonance and relevance to the public in their selection of content and mode of delivery^9^.

As such, there have been some attempts to shift from a reflective, information-rich way of thinking—typical of the early 2000s—to non-reflective or automatic modes more recently^9^. Often cited are government initiatives to embrace the concept of ‘nudge’, which alludes to the notion of an easy-to-follow intervention aimed at altering a behaviour but not at the cost of banning options or significantly altering economic incentives^10^.

In 2014, for example, Public Health England (PHE), ran the *Antibiotic Guardian* online campaign, reflecting ‘nudge’ theory by asking people to publicly pledge to avoid seeking an antibiotic for a non-serious infection when the next such occasion arose, effectively appealing to a social norm, and a herd mentality, for actions to be consistent with personal commitments^11^.

But importantly, communication campaigns are more likely to be optimised if carefully localised to specific population traits, norms and culture that underpin attitudes and behaviours towards antibiotic use and AMR. Typically, the universal, core, message of ‘misuse and overuse of antibiotics cause resistance’ is correct, informative and well-intentioned, but this does not reflect the diversity of access to antibiotics, prevalence of infectious diseases, healthcare systems, levels and diversity of AMR, socio-economic or socio-cultural differences, and multiple combinations thereof, that impact attitudes and behaviours towards antibiotics in different settings around the world.

Essentially, public and professional engagement around antibiotics and AMR, might benefit from content tailored to address specific local, and sub-population gaps in knowledge as well as the assemblage of influences on attitudes, responses and behaviours towards antibiotic use and AMR, while also ensuring antibiotic availability where access is restricted^12,13^.

## Communication for change—power of the prescriber, public–patient, or both?

While a 2022 WHO survey found that one third of citizens living in 14 (out of 27) European Union (EU) countries obtain antibiotics off prescription, in the UK and most EU countries, antibiotics are largely sourced on prescription-only which, to some extent, shifts the goal of messages aimed at public^14^. If prescribing power lies solely in the hands of healthcare professionals, then this might limit the size of the effect that can be achieved through asking public to take personal action (even in a scenario where the public was maximally responsive to direct requests for action).

As such, communication that does not directly promote action but raises awareness and is, ideally, embedded in the socio-cultural tenets of understanding around antibiotics, as well as being situated in relation to the dynamics and discourse of the medical encounter, might carry more weight.

UK primary care physicians acknowledge that antibiotic prescribing is often done under a range of pressures including—in their view—and ranked as the top concern according to a recent review by Borek et al., were beliefs about the consequences for patient and doctor of the antibiotic prescribing decision. Other leading concerns were social influences comprising knowledge and perceptions of the patient, and the physician’s perceptions of the patient’s expectations and satisfaction; communications skills and strategies; as well as time and workload^15^. Physicians also note that some patients still expect and even demand antibiotics^16,17^.

It follows that improving antibiotic stewardship efforts by healthcare professionals should go a long way towards reducing antibiotic use, and UK data suggest that since 2014, antibiotic prescribing has dropped, with exception for the anomalies in prescribing practice associated with the dramatic shifts across healthcare precipitated by the COVID-19 pandemic^18^.

But intrinsic to these leading concerns around prescribing by primary care physicians is the acknowledgement of the part played by the patient in the dynamics of the doctor–patient encounter. Pre-empting this doctor–patient negotiation about whether to prescribe antibiotics lies an individual who enters the consultation room with a lifetime of experiences, including those of health. In essence, a patient arrives with a story to tell, a richly textured piece of fabric woven from the threads of socio-cultural, familial, educational, generational, geographical, and ethnic influences of their personal history. These ingrained factors shape the conversation and not only have a bearing on the patient’s responses but also a clinician’s knowledge and perceptions of the patient, and the clinician’s perceptions of the patient’s expectations and satisfaction^6,19^.

In effect, to advance public engagement with AMR whether inside, or outside of, a medical consultation, patient or public, we need to appeal to the melange of influences that carve out our understanding and perceptions around antibiotic use and AMR, including on a pragmatic level, when and how to self-manage minor infections without antibiotics.

Here, in the remainder of this commentary, we advocate for storytelling as one potential way to embrace and encapsulate this plethora of values into a communication approach that fundamentally has universal resonance across all of our lives, across miles and across time.

## Storytelling—a tool to surface an individual patient’s rich and relevant background

In essence, it is this person–patient with a story to tell that offers an alternative or additional method of both research and communication around any chosen phenomenon—in this case, AMR. A story may be told during a medical consultation, in everyday conversation (in person or on social media, say) or in a more structured and professional format, for example, storytelling as performance or via digital media, termed digital storytelling (DST)—essentially entailing the creation of 3–5-min short films that combine photographs or video, voiceover narration, visual effects, music and other auditory means of expression.

To date, storytelling is an emergent method for both research and intervention purposes, and may take many forms including the telling of verbal or written stories, ‘Photovoice’ (use of participant-taken photographs and narratives to tell the story), plays, song, film, and art^20^.

Theories and thinking about storytelling are extensive and a single definition that captures the entirety of its essence is elusive; however, Jerome Bruner, a twentieth century psychologist known for exploring cognitive learning, is often cited for his description of storytelling as an ‘act of mind’, if storytelling is considered a way of thinking about the world^21^.

If business is about forming connections with customers – internal or external to a company, then storytelling has a successful history of use in this sector as a means of sharing experiences behind brands, advocates, customers, and employees that connect on an emotional level. Some similarities exist with storytelling as used in health and medicine, for educational, advocacy, therapeutic, or even research purposes. For example, in health promotion, storytelling has often found application in marginalised groups through providing a voice to those often overlooked. Young, socially deprived and isolated, Puerto Rican women with sexual health issues found group storytelling provided a safe space in which they reflected on personal memories, and found that storytelling instilled a sense of control over their health and future goals, as well as an opportunity to find connection, social acceptance and empathy among peers. Storytelling, partly through metaphor, can also source insights on how culture and history impact meaning in the health context. For example, storytelling to investigate antiretroviral therapies (ART) and HIV stigma in parts of South Africa has shown that their cultural history of apartheid and armed struggle impact the meaning given to compliance with ART such as battling to overcome HIV. Understanding the drivers of health-related behaviours can feed into education and advocacy campaigns^22^.

Storytelling as a research tool holds with phenomenological principles, in being a form of qualitative research that derives its value by drawing on an individual’s unique lived experiences. In contrast, the extensive corpus of evidence-based health and medical research is often based on a scientific epistemology, drawing on statistical tools to generate a set of results and conclusions that are considered generalisable to the wider population. The revered status of the randomised controlled trial is a case in point. As such, a possible limitation of storytelling is in a taking a largely arts-based method and using this to investigate research questions that reside within the health and medical arena, where it does not fit with the conventional scientific method of investigation. Another limitation, that manifests in practice, is given the labour-intensive nature of creating (digital) stories as a data gathering method, only a limited number of digital stories are created and there is inherent uncertainty around whether those stories created are sufficiently compelling stories and provide accounts of experiences rich enough for analysis and in generating the meaning required to answer a research question.

As an aside, but a pertinent one that helps elucidate its value, *storytelling* differs from *story*. Michael Wilson, storytelling academic from the Storytelling Academy, University of Loughborough, UK, explains the process involved in story*telling* in that, ‘… choices about storytelling alter the story’s reception and ultimately allow for meaning to be nuanced through the context in which it is told.’ Aspects such as the characterisation or switching the chronological order of events, the setting or the historical perspective all serve to alter the *telling* of the story, although the story itself usually remains essentially the same^21^.

As such, storytelling is framed as a way of negotiating the nuances of personal experience, embodied within person-patient storyteller at its heart. Such reasoning chimes with a theoretical perspective known as philosophical hermeneutics whereby we all, as humans, bring an historically embedded consciousness to our interpretation of a given phenomenon or situation, a perspective that applies to the doctor-patient encounter as much as any other human interaction^23^.

But the relevance of philosophical hermeneutics goes further, in that it proposes the concept of the ‘fusion of horizons’, where an horizon is ‘the range of vision that includes everything that can be seen from a particular vantage point’^23^.

This ‘fusion of horizons’ manifests through conversation or negotiation of ideas with another person, a text, an artwork, or the crafting and telling of a story, among other things including, to a certain extent, the negotiation inherent in shared decision-making about antibiotic prescribing between doctor and patient^24^. The latter finds echoes in the work of Rita Charon, who developed the concept of narrative competence in medicine, which is specific to optimising the doctor-patient consultation by nurturing a more ‘humane’ and empathetic understanding of a patient’s story and plight to complement the biological understanding of their case^22^.

Broom points out that ‘how AMR is made visible and knowable shapes not only how institutions respond to AMR, but also the meanings that are attributed to it by both communities of clinicians and laypersons’^6^.

We propose that storytelling, and specifically digital storytelling may offer a way of making AMR ‘visible and knowable’ to the public.

## Storytelling of the digital kind

Digital storytelling has a relatively recent history and has been used most extensively with marginalised populations and social justice, but also increasingly for educational, therapeutic and advocacy purposes within the public health landscape. Here, we support the use of digital storytelling as a participatory way to communicate (share, generate and create meaning) authentic lived experiences of AMR told in the first person.

Michael Lang, founder of CommonLanguage DST, Calgary, Canada, has made over 900 digital stories, primarily in health and wellness contexts. Referencing story theorist and Hollywood screenwriter, Robert McKee, Lang points out that ‘creative talent is the ability to connect two seemingly unrelated things to create a ‘third thing’ that is new or unknown,’ and in the process provide deeper insight into the phenomena being explored^25^.

Lang advocates for a structured and participatory digital storytelling process—finding, telling, crafting, sharing—that can facilitate the creation of a meaningful ‘third thing’ and as such, allow new understanding to emerge from of the health/disease experiences of patients and their families. However, although digital storytelling follows this structure, facilitating a meaningful digital storytelling experience within a health care context has an essential form but does not follow a formula. Rigidity would stem the flow and cultivation of a rich and meaningful end product^25^. Learning how to support patients and families to tell their stories, he says, is how healthcare can both leverage the generative power of storytelling to enhance understanding but also communicate important health messages, incorporating often hard-to-access values and influences, in a more compelling manner.

This process may reveal insights not only to the storyteller about their lived health experience, but that resonate with a listener or viewer with a relevance and meaning possibly untapped through other means of communication. Fundamentally, storytelling helps us to make sense of our thoughts and experiences, our interactions with each other and our environments, and this process helps to formulate our beliefs, identities and values^21^. In this way (digital) storytelling may partly address some of the shortfalls of historical or existing methods of communicating concepts of AMR, and this forms the basis to the research project, entitled StoryBug outlined below.

If digital storytelling aims to stimulate intentional, and ultimately behavioural change, it is unlikely to satisfy this goal as a stand-alone intervention in the real world. A carefully constructed communication campaign may incorporate storytelling into the mix that might include semi-structured interviews, focus groups, or short narrative videos shown on tablets for example.

StoryBug is a project led by researchers at University College London (UCL), which is using this digital storytelling process with people who have personal experiences of AMR to express and generate meaningful significance around what has happened to them. Digital storytellers choose the key ‘moment’ of their story that holds greatest meaning for them, and they build their story with this as a focal point, using personally selected photographs, music, art, voice over, script that illustrate and add value to their experience. It is precisely these values of storytelling, that render the story rich with meaning and resonance both to the teller and the listener. In this way, digital storytelling in the StoryBug project may potentially fulfil, at least in part, the gaps left by other more conventional methods of communication drawing on the biopsychosocial underpinnings of AMR.

StoryBug endeavours to evaluate the communication value of the digital stories created through a screening with the general public and possibly a small number of clinicians. It is hoped that digital storytelling in relation to AMR may also find application in the creation of stories with clinicians, and moving beyond the direct human element of AMR to gathering digital stories from vets, farmers, and people involved in environmental aspects of this interconnected, One Health issue.

Turning once more to Lang who cites from Rachel Naomi Remen’s book, Kitchen table Wisdom. He recounts that, ‘Facts bring us to knowledge, but stories lead to wisdom.’ We have facts, and undisputedly we need more, but if stories lead to wisdom, then (story)tell me more.

## Data Availability

There are no new data of relevance in this article to make available.
